# Ten Simple Rules for Developing a Short Bioinformatics Training Course

**DOI:** 10.1371/journal.pcbi.1002245

**Published:** 2011-10-27

**Authors:** Allegra Via, Javier De Las Rivas, Teresa K. Attwood, David Landsman, Michelle D. Brazas, Jack A. M. Leunissen, Anna Tramontano, Maria Victoria Schneider

**Affiliations:** 1 Biocomputing Group, Department of Physics, Sapienza University of Rome, Rome, Italy; 2 Bioinformatics & Functional Genomics Research Group, Cancer Research Center (IBMCC, CSIC/USAL), Salamanca, Spain; 3 Faculty of Life Sciences and School of Computer Science, University of Manchester, Manchester, United Kingdom; 4 Computational Biology Branch, National Center for Biotechnology Information, National Library of Medicine, National Institutes of Health, Maryland, United States of America; 5 Informatics and Bio-computing, Ontario Institute for Cancer Research, MaRS Centre, Toronto, Canada; 6 Laboratory of Bioinformatics, Wageningen University, Wageningen, The Netherlands; 7 Outreach and Training Team, EMBL Outstation, European Bioinformatics Institute, Wellcome Trust Genome Campus, Hinxton, United Kingdom

## Introduction

This paper considers what makes a short course in bioinformatics successful. In today's research environment, exposure to bioinformatics training is something that anyone embarking on life sciences research is likely to need at some point. Furthermore, as research technologies evolve, this need will continue to grow. In fact, as a consequence of the introduction of high-throughput technologies, there has already been an increase in demand for training relating to the use of computational resources and tools designed for high-throughput data storage, retrieval, and analysis. Biologists and computational scientists alike are seeking postgraduate learning opportunities in various bioinformatics topics that meet the needs and time restrictions of their schedules. Short, intensive bioinformatics courses (typically from a couple of days to a week in length, and covering a variety of topics) are available throughout the world, and more continue to be developed to meet the growing training needs. The challenges, however, when planning, organising, and delivering such courses, are not trivial [1], especially considering the heterogeneous backgrounds of participants. Here, we address such challenges and present a consensus of rules derived from the shared expertise of several bioinformatics trainers. While the rules apply broadly to bioinformatics training, aspects addressing specific audiences are also discussed in order to make these rules pragmatic and applicable to a wide range of readers. Delivering bioinformatics training is both crucial to facilitate the use of, and to exploit the investment in, bioinformatics tools and resources, and an excellent opportunity to solicit user evaluation and feedback to improve them. One point of crucial interest to the training course community concerns material preparation and distribution. Preparing effective materials (slides, notes, references, etc.) entails a huge effort that would be enormously facilitated if course developers could start from a body of available materials, for example if they could gain access to repositories of materials deposited by trainers of other courses. This was one of the reasons motivating the Bioinformatics Training Network (BTN) to set up the BTN website (http://www.biotnet.org/), which has been planned as a vessel for the training community to share and disseminate course information and materials. Course developers are warmly welcome to subscribe to the site and make available their materials to the community [2].

## Rule 1: Set Practical and Realistic Expectations

It is critical to explicitly identify the training objectives and expected outcomes from the outset. Begin by devising the title of your course and specifying the target audience (e.g., laboratory biologists, computational scientists). This information is not only useful for trainers to help appropriately focus and weight the contents of their training sessions, but is also vital for participants. By explicitly stating the course objectives up front, trainees are better oriented to the expected outcomes and are more likely to be satisfied with the course. As most training sessions are based on slide presentations, dedicate at least one slide (preferably, while providing the session overview) to the learning objectives, and mention how these will be achieved, using specific examples whenever possible; if appropriate, also mention how the knowledge gained and skill set(s) will be useful for trainees' work environments. Stating what participants will not learn to do (e.g., to avoid over-estimation of the depth of analysis that can be achieved in a short course) is also important for tempering their expectations.

## Rule 2: Verify That Trainees' Expectations Match Course Scope

Verify that trainees' expectations match what will be delivered. The most effective mechanism to ensure that expectations are well matched is to collect information from trainees prior to the training session itself (e.g., via a questionnaire), or by discussions with trainees at the start of the course. Obtaining such information early on allows time to alter course materials to better meet participant expectations, for example by adjusting case studies and examples to reflect the audience's interests. Furthermore, this will make you aware of the trainees' different backgrounds. Read, or listen to, and evaluate all responses, both to discern whether the course content matches participant expectations and to learn what the trainees' needs are. Such information will also allow you to detect clusters of trainees: e.g., those working with a particular model organism, those more interested in DNA than in proteins, or more plant than animal scientists. Useful information to collect includes their research backgrounds and computational skill sets, their current projects relevant to the course, and their expectations of the training (e.g., what reasons led them to apply for this particular course?). Also solicit information from trainees about the biological problems they wish to solve by participating in the course.

## Rule 3: Plan Exercises and Activities and Test Resources before Delivery

Plan the course in independent units/modules, each with an introduction, set of aims, list of actions, and potential difficulties. When a new module is introduced, recall the achievements of the previous module, and state what tasks participants will be able to additionally accomplish at the end of the new module.

If you, the trainer, are also responsible for the resource/tool being presented, you are likely to be able to handle unexpected queries or problems. However, many trainers deliver sessions on resources/tools built and maintained somewhere else by someone else, using someone else's data. Regardless, always prepare an alternative plan in anticipation of unforeseen difficulties. For example, at short notice, you might not be able to use live queries, so ensure that you have sufficient back-up material (e.g., animations, videos, etc.) to allow you nevertheless to deliver your training session effectively.

To appear as prepared and experienced as possible, try your practical exercises beforehand. In cases where the query or task required to a bioinformatics server takes a long time, or is too demanding on the service provider, either begin with smaller query datasets, or provide the task results after trainees have prepared the query set-up, so that they still gain the experience of performing the task and class time is used more efficiently. It is important to note that some service providers will often hold query results for 48 hours.

## Rule 4: Ensure Computational Equipment Preparedness and Hands-On Support Availability

Ensure (or rather, insist) that workstations (Linux, Mac, or PC) have all the necessary software installed to allow trainees to complete the course. Make sure that the venue provides each trainee (or, at most, each pair of trainees) with one computer. Where trainees are required to bring their own workstation (e.g., laptop), provide enough instruction and test commands to ensure that software and dependencies have been properly set up ahead of time. Request that a system support technologist be available, and in the room, when starting your sessions, to ensure the functionality of the classroom workstations and/or of the participants' personal computers.

Do not underestimate the trainer/trainee ratio, especially in consideration of the trainees' diverse backgrounds. Be prepared to provide extra hands-on support while trainees become familiar with new interfaces, tools, and resources. Such support may be provided by trainers of other modules, tutorial assistants, past trainees, or even current trainees who are familiar with the tool/resource basics.

## Rule 5: Use the Dynamic World of Bioinformatics Resources and Tools as a Learning Opportunity

Provide time references for the information you deliver, as bioinformatics resources and tools, and stored data, evolve continuously. Place emphasis on the “official” sites, as these are most likely to remain stable reference points for trainees. When creating your materials and exercises, as much as possible, avoid screen-shots, as these date quickly—otherwise, you risk spending substantial amounts of time updating outdated slides rather than concentrating on developing suitable case studies and examples relevant to your audience. Describe the essence of data that can be retrieved from a particular resource and the principles governing a tool, rather than sticking to specific releases, web interfaces, or, for example, to tables of ranked results, which are likely to differ from day to day, as new data become available in the databases. Take into account that new data may have been added to the databases you are planning to use, and hence the outputs of the queries might be different from those you planned to demonstrate. As this occurrence is actually an integral part of bioinformatics, this can be beneficial for trainees to witness—you might even want to explore such situations extensively, to convey the idea that resources and tools are dynamic.

## Rule 6: Balance Concepts with Practical Outcomes

Bioinformatics training encompasses a vast amount of learned skills. Acquiring these skills is a bit like learning to ride a bicycle, where it is best to just start pedalling, because watching others will not help you learn the process! Of course, it is important to provide trainees with the fundamental concepts and theoretical background to ensure that they can use bioinformatics tools and resources meaningfully. Nevertheless, it is a good rule to provide a balance between the theoretical/technical and contextual aspects. For example, many trainees may not value information on flat-files, relational schemas, APIs, and web services, but will be more concerned about knowing which tools and resources to use for their specific needs, and why, and how to interpret their outputs (just as the average cyclist is not interested in the internal workings of the gearbox, as long as they know how and when to shift gear!). Discuss the limitations of the methods without getting carried away by the intricacies of the algorithms or the minutiae of a tool's capabilities. Ensure that you cover not only those questions that bioinformatics approaches can answer, but also the limitations of bioinformatics, explicitly illustrating examples that cannot be answered.

Avoid long sessions of browsing around web interfaces or showing one screenshot after another. Trainees will be eager to try tools themselves and will benefit far more from a well-planned session, with adequate time allocated to an exercise or simple exploration, than from merely watching someone else explore for them. When giving a demonstration, try to get participants to follow along with you. To compensate for the likely diversity in speed and computer-ease of your audience, when possible, pair trainees of different backgrounds together and progress activities at a speed that will allow all trainees to keep pace. Once you have completed a task, confirm that everyone has achieved the result, and recapitulate the scope of the actions to reinforce the meaning and significance of the session. If you allow trainees to work by themselves on specific tasks, conclude with what you expected them to have achieved and how! Also consider providing this summary of steps and expected outcomes in an electronic/paper version as an addendum, as trainees might want, and would certainly benefit from being able, to review the task again, on their own time. Furthermore, trainees will often be eager to share what they have learnt when they return to their work environments, so having a set of good course manuals/practical exercises is essential to enable them to do so. Absolutely avoid spending 80% of the session talking and then rushing through the last 20% of the practical aspects. Moreover, try to avoid telling trainees to finish later (on their own) whatever they did not complete, as they will probably not do so, will feel resentful because what they really wanted to do was not done and, more importantly, they will have lost the important recap and reinforcement that you can provide.

## Rule 7: Reinforce Learning with Contextual and “Real World Experience” Examples

Wherever possible, provide appropriate biological context: examples without relevant context lack meaning and fail to engage trainees. After introducing a new concept, allow time to put the concept immediately into action. Begin hands-on exercises with a short worked example where everyone can complete contextual learning on a common dataset. Follow this with time for further exploration: here, you might either provide a second dataset or, if relevant or practicable, invite trainees to use their own. If appropriate, illustrate examples taken from your real world research experience. For instance, outline biological problems that you tackled with bioinformatics and describe resources and tools that you adopted to solve them and to achieve your findings and how.

## Rule 8: Ensure the Methods/Tools Have Relevance to the Trainee Experience and Scientific Research Needs

Design your materials such that the examples you provide illustrate the concepts you wish to convey and, at the same time, are relevant to the research interests of at least some of the trainees. Whenever prior information about trainees' interests is available, use it. Appreciate that a plant biologist will not have a need for human-centric examples, nor will they find them comparable. The more relevant you make the examples for the trainees, the more likely they are to retain their interest and develop their skills! Furthermore, encourage trainees to explore the tools and resources presented during the course not only with the carefully prepared examples provided, but also from the perspective of their own research interests: nothing motivates as much as the need to solve one's own problems!

The use of tools and resources from the perspective of personal research interests, will lead new users to take a fresh critical look at them. From this perspective, trainees might be able to provide a special assessment of the tools and resources introduced in the course which would be different and complementary to the one that experienced users can provide. Trainers can gain an understanding of how easy (or hard) exploring web interfaces or programmatically access and parse resources is, and specific comments on what is intuitive or not to trainees can be captured informally or formally (e.g., through surveys). In this regard, you may explain to trainees that evaluation and feedback collected during the actual training course or in a final feedback survey can aid significantly to improve bioinformatics resources.

## Rule 9: Allow for Interactivity and Provide Time for Reflection, Individual Analysis, and Exploration

Ensure interactivity and time for reflection. Provide time for trainees to acquaint themselves with the interfaces of the tools/resources, and to understand their contents: allowing trainees to explore a tool or resource on their own tends to promote greater retention of concepts.

Schedule 10–15 minutes at the end of each module to review the presented concepts, and to stimulate questions from the trainees, who will probably have only just started processing the information.

Do not simply rely on a set of slides and step-by-step tutorials to teach concepts. Make use of flip-charts to brainstorm together, asking trainees for ideas and alternative ways to resolve particular biological questions. Group sessions like this, where trainees are encouraged to share their thoughts and views with the whole class, can help both to identify common issues and aspects to be explored, and to highlight any trainee limitations and/or mismatched expectations. Moreover, incorporating such group discussions directly into training sessions can often help to instil a greater level of understanding than when trainees are left to passively explore set examples (or to copy and paste scripts with no explanation of what these might achieve). Exploit such brainstorming sessions to demonstrate how bioinformatics tools and resources can help to address, and sometimes solve, complex problems.

Depending on the time available, include quizzes and/or problem-solving tasks and open discussion sessions in which participants can reflect on the skills they've learned and how these might be used to address questions of interest to them.

Provide trainees (perhaps in pairs or groups) with a brief set of questions prior to, and after, the training course. Questions that probe their knowledge and understanding of bioinformatics are useful both for trainers (to verify that the course has been pitched correctly and to establish what knowledge has been gained) and for trainees. Furthermore, by asking trainees to think about, and answer, a series of course-relevant questions, you ensure adequate time for concept and content digestion and reflection.

## Rule 10: Encourage Independent Thinking and Problem Solving

Finally, teach to fish rather than give fish! In other words, try to develop independent thinking rather than simply spoon-feeding trainees with slides and step-by-step tutorials: it is more important to learn how to tackle research questions with bioinformatics, and to know where/how to search for solutions, than it is to learn about the minutiae of every available tool and resource.
